# Supplementary material for: CoPheScan: phenome-wide association studies accounting for linkage disequilibrium
Source: Nat Commun. 2024 Jul 12;15:5862. doi: 10.1038/s41467-024-49990-8 (PMC11245513; doi:10.1038/s41467-024-49990-8)
Supplement: Supplementary file 3 — Description of Additional Supplementary Files [file 41467_2024_49990_MOESM3_ESM.pdf]

Description of Additional Supplementary Files

File Name: Supplementary Data 1
Description: Hc.cutoff determination using FDR in simulated data.

File Name: Supplementary Data 2
Description: UKBB phenotypes used as query traits.

File Name: Supplementary Data 3
Description: Additional phenotypes used for the analysis of TYK2 variants.

File Name: Supplementary Data 4
Description: Query variants identified in the FinnGen dataset. Mapped table: where variants of the FinnGen primary traits are mapped to equivalent UKBB traits.

File Name: Supplementary Data 5
Description: Query variants identified in the FinnGen dataset. Unmapped table: where no equivalent traits in UKBB were mapped to FinnGen primary traits or where r_g information was not available.

File Name: Supplementary Data 6
Description: Query variants identified in the pQTL dataset from Ferkingstad et al., 2021

File Name: Supplementary Data 7
Description: Query variants from the UKBB dataset which are protein truncating variants with MAF > 0.001.

File Name: Supplementary Data 8
Description: UKBB traits with high Hc with the variants from the FinnGen dataset.

File Name: Supplementary Data 9
Description: UKBB traits with high Hc with the variants from the pQTL dataset.

File Name: Supplementary Data 10
Description: UKBB traits with high Hc with the variants from the protein truncating variants dataset.

File Name: Supplementary Data 11
Description: UKBB and GWAS catalog traits with high Hc with the TYK2 and SLC39A8 variants.

File Name: Supplementary Data 12
Description: Priors inferred from real data.
